# Weathering Effects on Concrete Objects Based on 3D Scanned Examples and Geometric Features

**DOI:** 10.3390/s23218979

**Published:** 2023-11-05

**Authors:** Sang Il Park

**Affiliations:** Department of Software, Sejong University, Seoul 05006, Republic of Korea; sipark@sejong.ac.kr

**Keywords:** computer graphics, geometry synthesis, 3D scan, example-based, weathering

## Abstract

In this paper, we introduce a method for simulating the deformation of concrete surfaces due to weathering employing an example-based approach to replicate shape changes observed in real-world objects. A key challenge in implementing this approach is the scarcity of opportunities to measure shapes both before and after the weathering process. To overcome this limitation, we utilize concrete bricks collected from real-world environments as standardized examples, allowing for an analysis of erosion. By measuring erosion based on the estimated original shape, we correlate the characteristics of erosion with geometric features such as curvature and accessibility. We then apply this analysis to simulate new weathering effects in a given input model in alignment with its own geometric features. Our method yields visually compelling results while reproducing the variation of geometric weathering effects.

## 1. Introduction

Time affects everything, including architectural structures and human-made monuments. As a result, weathering has become a significant aspect in enhancing the realism of 3D virtual objects, with extensive research conducted over decades to provide artists with tools for generating such effects. This body of research spans from 2D texture synthesis to 3D geometry generation [1]. The signs of weathering are particularly noticeable in aged buildings and structures, emphasizing the importance of this process in our daily lives. Concrete, a material used in construction for over a thousand years, is prevalent in our surroundings. Consequently, the modeling of concrete wall or object weathering or fracture is vital for creating convincing 3D scenes.

In this paper, we propose a method for synthesizing the effect of weathering on 3D concrete objects, distinguishing our work from previous studies through an example-based approach. This method reproduces weathered 3D geometries through a process guided by the original geometry. The development of an example-based method necessitates understanding the weathering process, requiring the measurement of shapes before and after weathering. Recent research conducted in controlled laboratory settings, such as the measurement of iron rusting, has led to the synthesis of 3D models like those presented in [2,3]. However, applying these methods to materials like concrete is challenging due to this material’s prolonged weathering time.

To facilitate the analysis of erosion, our main idea is to take weathered concrete bricks from the real world as examples. While brick dimensions can differ, there exist standardized sizes prevalent within certain regions that enable us to infer the original measurements of the weathered bricks. In addition, as bricks are widely used in construction, samples reflecting different weathering levels are readily available. We collect and scan multiple samples, ranging from new to heavily aged and damaged, building a database with which to analyze erosion characteristics. During the synthesis process with a given input 3D model, we generate a new weathered geometry by replicating the erosion pattern in 3D space, reflecting an object’s original shape. It is worth noting that we focus solely on geometric changes during weathering, without considering color changes, and assume that bricks always lose volume through erosion.

The main contributions of the proposed method are as follows:

- The introduction of an example-based approach to simulating weathering effects on concrete objects, drawing from 3D scans of weathered bricks.
- The adaptation of texture synthesis frameworks for 3D geometries, with curvature and accessibility metrics serving as key factors for enhanced realism in weathering generation.
- The ability to synthesize complex weathering patterns on user-provided 3D models, demonstrating our technique’s robustness and versatility.

This paper is organized as follows: In Section 2, we discuss related work. Section 3.1 offers an overview of our method. Section 3.2 details the construction of an example brick database and analyzes its erosion, while Section 3.3 presents our synthesis method, explaining how the geometric features of the input model were incorporated. Finally, Section 4 provides our experimental results, and Section 5 concludes the paper by discussing our method’s advantages and limitations.

## 2. Related Work

Applying lifelike weathering effects to textures and surfaces is pivotal for generating convincing virtual environments, a need that is particularly urgent in the film and gaming industries. While the demand is high, the current methods often involve laborious manual work performed by specialized artists. Over the last twenty years, researchers have been keen to simplify this process, as highlighted in the seminal surveys conducted by Merillou and Ghazanfarpour [1] and a comprehensive book by Dorsey et al. [4]. Generally, approaches to this issue fall into one of two categories: simulation-based or example-based weathering. Although our review is not exhaustive, we discuss key methods from both categories.

Simulation-based approaches typically employ user-defined parametric rules or phenomenological models to simulate weathering patterns. For instance, Becker et al. introduced a rule-based model for generating surface imperfections [5]. In the late 1990s, Dorsey and colleagues published pioneering works on the phenomenological simulation of weathering, addressing topics like patina generation on metal surfaces [6], flow patterns [7], and volumetric stone weathering [8]. Jones et al. later introduced an effective method for weathering concave stone surfaces [9]. Both these latter studies, refs. [8,9], are quite similar to ours in that they utilize a volumetric representation for erosion calculations. However, our approach diverges by offering the capacity to learn from real-world examples rather than relying solely on simulations. In more recent developments, the research by Ishitobi et al. [10] and Verhulst et al. [11] presented techniques for simulating weathering effects on metal and concrete surfaces, respectively. However, these approaches also differ from ours, primarily due to their reliance on physical simulations.

Example-based methods draw upon real-world samples to produce realistic and detailed effects. Wang et al., for instance, computed appearance attributes based on a single weathered object photo to estimate the degree of weathering per pixel and transfer this effect to new objects [12]. Xue et al. extended this approach to adapt to varying illumination conditions [13]. Similarly, Bosch et al. optimized simulation parameters to recreate weathering appearances that closely matched those of example photos [14]. Some techniques use multiple photographs to capture time-variant appearances, such as those developed by Lu et al. [2,15] and Gu et al. [3]. Our method differs in that, unlike other example-based approaches, which mainly focus on textures, we concentrate on the geometric transformations caused by weathering.

From our perspective, understanding geometric loss due to erosion is also relevant to our work. In this context, studies on fracture phenomena are also highly relevant. There are various simulation-based methods used in computer graphics addressing brittle [16] or ductile [17] fractures, including those developed in the work by O’Brien and colleagues. Iben and O’Brien, for example, generated a variety of surface crack patterns through physical simulation [18]. More recently, Glondu et al. presented an example-based method for generating fractures based on photographs [19], wherein simulation parameters are optimized using a single fracture sample photograph to produce similar-looking fractures on arbitrary input objects.

## 3. Materials and Methods

### 3.1. Method Overview

We adopted an example-based strategy to simulate weathering effects on 3D concrete objects. The cornerstone of this methodology is securing paired shape examples both prior to and following the weathering process. Given that weathering is a time-consuming process, it is uncommon to find such sets of examples. To address this, we utilized concrete bricks for our examples. Their standardized dimensions enabled us to approximate their pre-weathered states.

**Figure 1:**
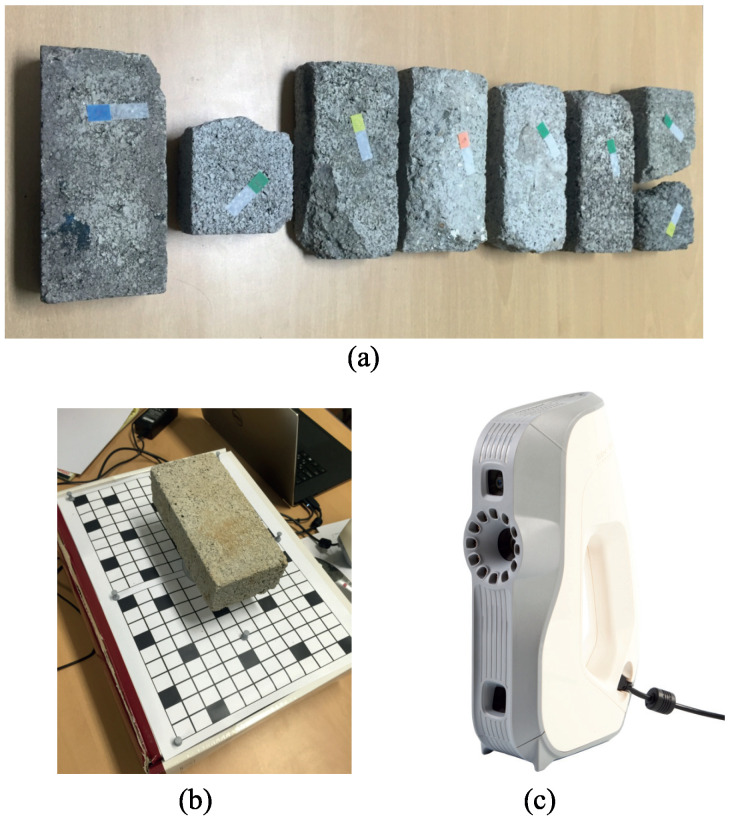
Data Collection: (**a**) collected weathered concrete bricks; (**b**) 3D scanning procedure; (**c**) Artec3D Eva 3D scanner.

**Figure 2:**
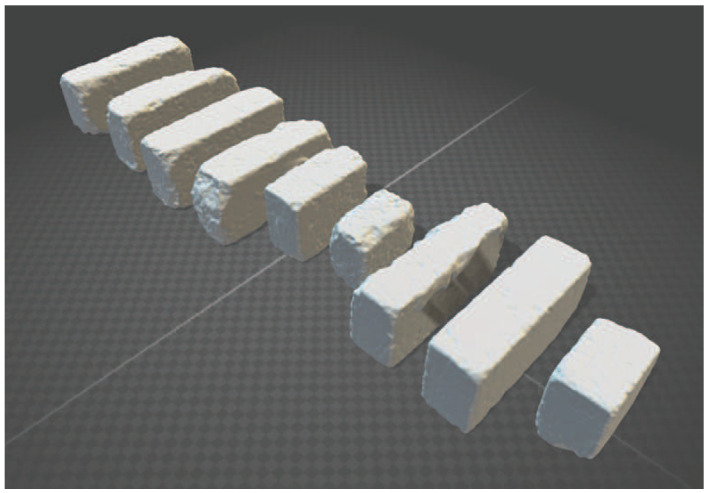
Scanned brick models.

We selectively collected weathered concrete bricks that had naturally undergone long-term weathering processes, avoiding those affected by artificial damage. These samples were then subjected to 3D scanning, as shown in Figure 1. For this purpose, we used an Artec3D Eva scanner by Artec Co., Inc. (Tokyo, Japan) due to its 0.5 mm accuracy level, which was sufficient for our measurement needs. The resulting mesh models comprised approximately 15,000 vertices and 30,000 triangles. We emphasize that our primary focus is on geometric alterations; thus, color information was not obtained. Figure 2 shows the scanned brick models.

**Figure 3:**
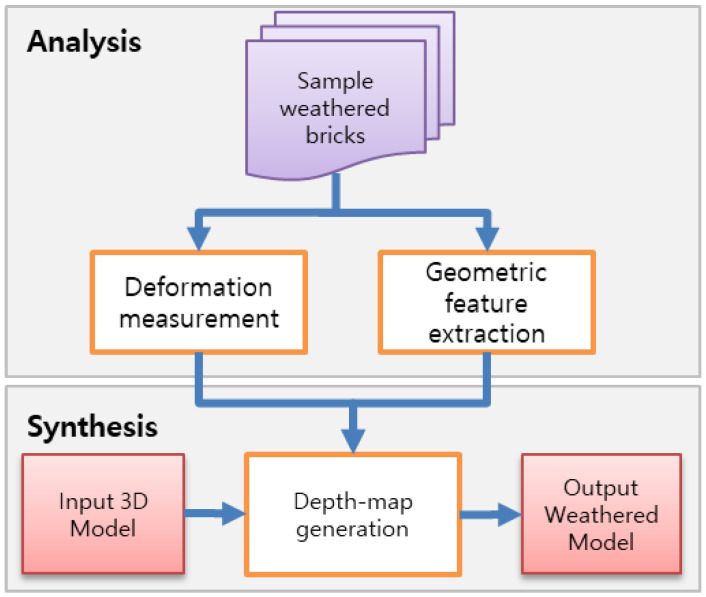
Overview of the method, consisting of two primary stages: weathering analysis and weathering synthesis.

Our approach consisted of two primary stages, namely weathering analysis and weathering synthesis, as illustrated in Figure 3. In the analysis stage, we quantified the changes due to weathering for each brick sample by cross-referencing them with their standard dimension specifications. To facilitate this process, we constructed a rudimentary 3D template mesh model conforming to a brick’s standard dimensions and computed the geometric deviations between this template and the scanned models. Due to the properties of concrete, its weathering effects are restricted to volume loss. We measured this volume loss and mapped it onto the corresponding regions on the surface of the template model. Additional geometric attributes, such as curvature, were also assessed to further characterize the deformations.

**Figure 4:**
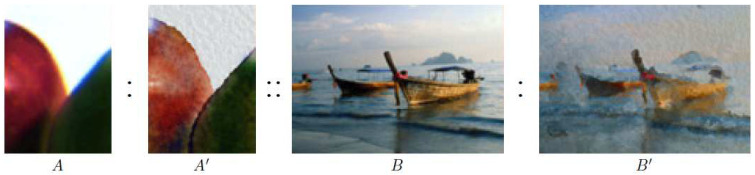
Image analogy [20]: Given a source image *A* with its filtered version $A^{\prime }$, the algorithm generates a new filtered target image $B^{\prime }$ from a given unfiltered target image *B* (images provided courtesy of [20]).

In the synthesis stage, we employed a 3D mesh model as the target input and generated weathering effects on it. Inspired by the image analogies framework developed by Hertzmann et al. [20], we adapted their algorithm to our weathering simulation. In their approach, as illustrated in Figure 4, a pair of unfiltered and filtered source images, along with an unfiltered target image, is used to create a new filtered target image. The core principle of their approach is to locate the best-matching patches between the source and target images to maintain existing symmetries. Applying this to our scenario, we designated the 3D brick template as the unfiltered source model and the scanned weathered bricks as the filtered versions. This enabled us to utilize the same framework to create a weathered version of a specified 3D target model.

### 3.2. Weathering Analysis

#### 3.2.1. Deformation Measurement

**Figure 5:**
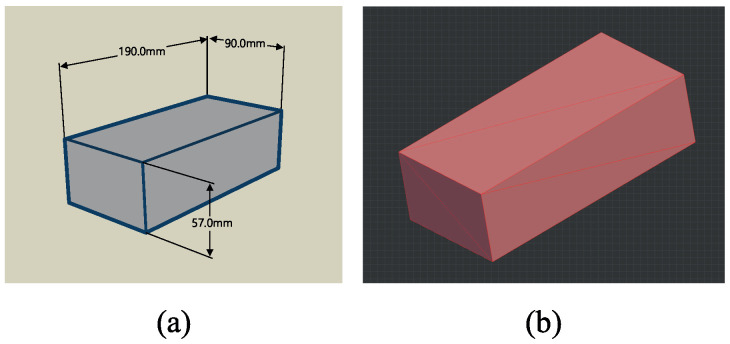
(**a**) Dimensions of the standard brick; (**b**) the template model.

To quantify the loss in volume due to weathering, we employed a template 3D brick mesh model constructed in accordance with standard specifications, as depicted in Figure 5a. We then compared this template with each of our scanned 3D models to evaluate the deformation. As shown in Figure 5b, the template mesh model’s faces were subdivided to match the vertex count of the scanned models.

Before conducting the measurement, each scanned 3D brick model was aligned with the template. We initially manually adjusted the global orientation and translation to approximate alignment, followed by fine-tuning using the Iterative Closest Point (ICP) algorithm [21]. Although various advanced surface-fitting techniques exist, the ICP method sufficed for our purposes due to the manual nature of our initial setup and the relatively straightforward geometries involved.

We determined volume loss by calculating the distance between the scanned model and the template. For this, we utilized ray-tracing and depth-mapping techniques. We cast rays from each point on the template’s surface in the opposite direction of its normal surface until it intersected with the scanned model.

**Figure 6:**
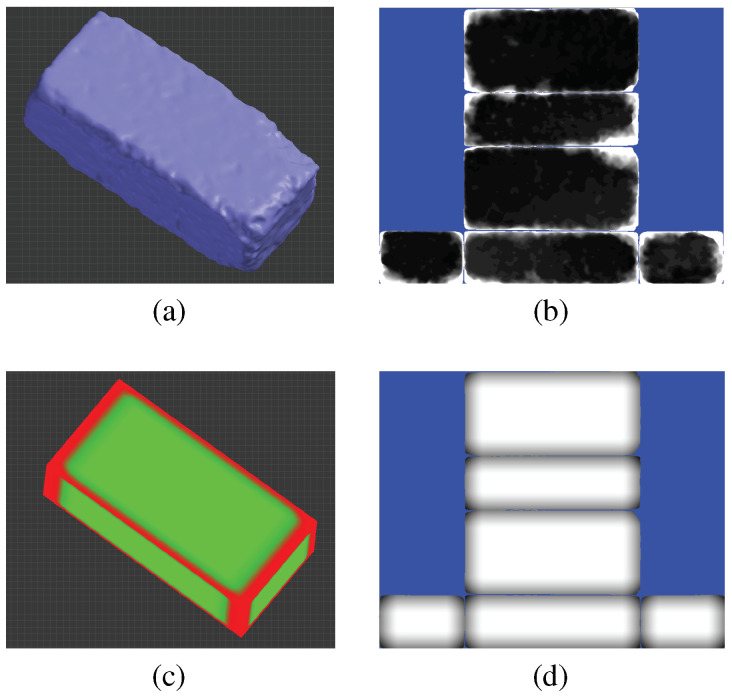
(**a**) Scanned weathered brick; (**b**) depth maps captured from six directions, consolidated into a single depth map; (**c**) smoothed curvature of the template model, with green indicating areas of low curvature and red signifying regions of high curvature values; (**d**) template-derived curvature map used to guide depth mapping.

The distance to this intersection point quantifies the deformation at that location, and these measurements are stored in a depth map. Using this approach, we compiled six depth maps for each scanned model, corresponding to each of the template’s rectangular faces. Figure 6a,b display a sample brick and its associated depth maps. The depth values were normalized and quantized to integer values in the [0, 255] range for pixel brightness representation. To maintain scanner accuracy, each pixel’s width in the depth map equates to 0.5 millimeters. Throughout this procedure, we generally assumed that the scanned models did not exhibit significant concavity, which held true for our dataset.

#### 3.2.2. Analysis of Geometric Features

Weathering effects vary across different locations for a single brick. For example, certain areas of a brick may exhibit more weathering than others. Several factors can contribute to these variations, including the object’s shape, exposure to environmental elements, and other ambient conditions. However, due to the scope of this study, we focused solely on geometric features that can be directly measured from the 3D model.

Despite a brick’s fundamentally simple geometry, it still exhibits varied weathering patterns across different areas, such as flat surfaces, edges, and corners. Therefore, we chose curvature as the distinguishing geometric feature for our analysis. We calculated the curvature at each vertex of the template brick model—representing the pre-weathered shape—and later correlated these values with the weathering patterns observed in the post-weathered, scanned brick models.

Given the simplicity of a rectangular parallel-piped model, its curvature is highly concentrated at the edges and vertex corners. To convey the influence of these high-curvature areas more broadly, we diffused the curvature values to neighboring vertices through a spreading process. This enabled us to capture more nuanced impacts near areas of high curvature.

The calculated curvature values are stored in an image format, termed the *curvature map*, analogous to the previously described depth map. Figure 6c,d display the 3D curvature values of the template model and its corresponding curvature map, respectively.

### 3.3. Weathering Effect Synthesis

Leveraging the insights from scanned 3D weathered brick models, we aimed to synthesize weathering effects on a new target model. We employed a texture synthesis framework to tackle this problem. Specifically, the image analogy method proposed by Herzmann et al. [20] served as a suitable basis for our approach, as outlined in Section 3.1.

**Figure 7:**
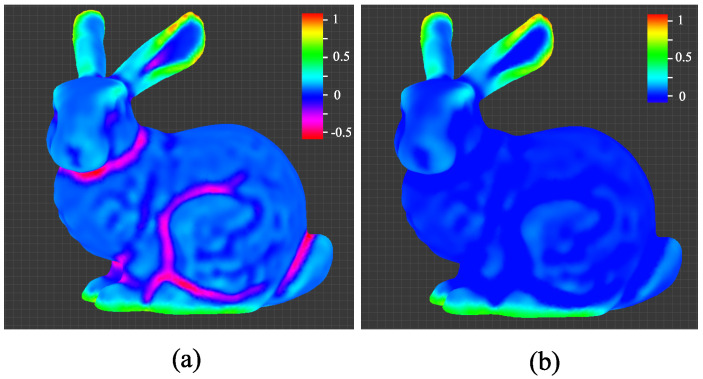
(**a**) The mean curvature values range from negative to positive, and the values are rescaled so that the maximum is 1. (**b**) Negative values, which were clamped and normalized.

To guide the process of applying weathering effects to the target model, we utilized geometric features, particularly mean curvature values [22], due to their ease of implementation. Figure 7a illustrates the computed mean curvature on the surface, where red and purple indicate areas of positive and negative curvature, respectively. Given that areas with positive curvature are more susceptible to weathering, we clamped the negative curvature values to zero and normalized the remaining positive values, thereby constructing a guiding scalar field. The resulting curvature field is displayed in Figure 7b.

**Figure 8:**
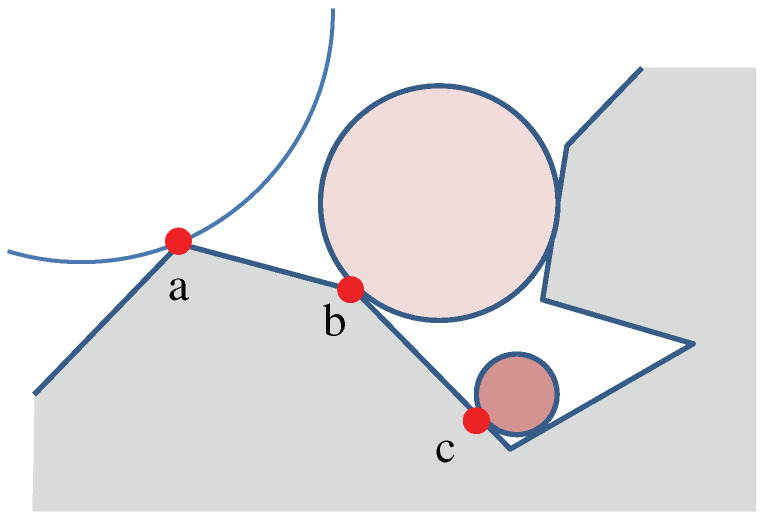
Accessibility measures the maximum radius of a circle that can touch a point without intersecting the surface: (**a**) presents infinite accessibility, while (**b**) shows higher accessibility than (**c**), reflecting its surroundings.

However, curvature alone may not fully encapsulate a surface’s exposure to weathering. To address this, we incorporated the concept of accessibility, as defined by Miller [23]. Accessibility measures the degree to which a point is exposed to its surrounding environment, as shown in Figure 8. We normalized these values to fit within the range of [0,1], thereby generating a complementary guide field.

**Figure 9:**
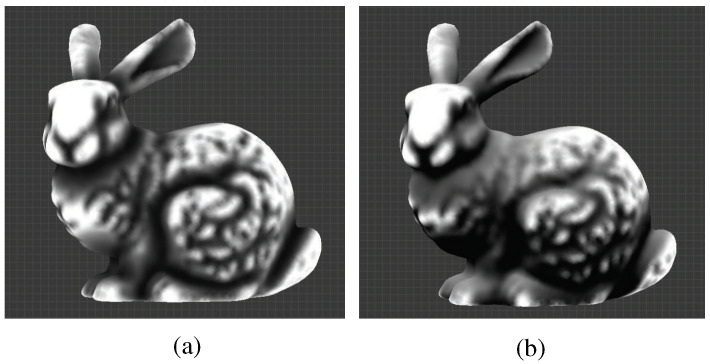
(**a**) The original accessibility; (**b**) the accessibility altered to emphasize the upward direction.

To account for the orientation of surface facets, we also introduced a directional component to our accessibility measure. Specifically, we modified the accessibility values by multiplying them by the dot product of the surface normal $n_{p}$ and the upward direction vector up as follows:

(1) $$\varepsilon_{p}^{*}=\varepsilon_{p}\left(n_{p}\cdot \mathrm{up}\right),$$

where $\varepsilon_{p}$ and $\varepsilon_{p}^{*}$ denote the original and modified accessibility values, respectively. Figure 9 contrasts the two sets of accessibility measures.

In summary, our method uses both curvature and accessibility as guide fields for weathering synthesis. Since our example bricks only provide curvature fields, we generated a third guiding field from the blurred depth maps for correlation with accessibility. To execute this synthesis, we adopted Turk’s “Texture Synthesis on Surface” technique [24] using local coordinate systems defined by the principal directions on the surface.

## 4. Results

**Figure 10:**
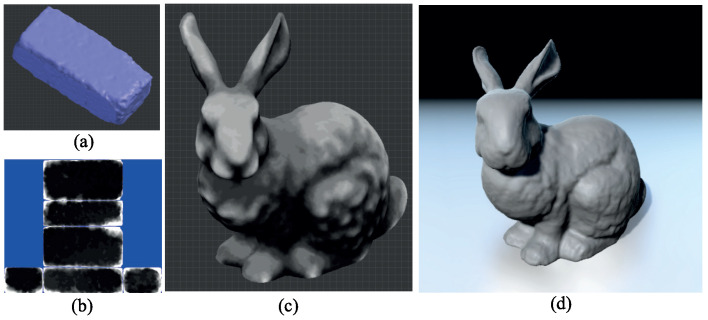
Weathered bunny model generated using a single example brick: (**a**) sample example brick; (**b**) sample depth map; (**c**) synthesized depth map; (**d**) final result after applying the depth map to the model’s vertices.

**Figure 11:**
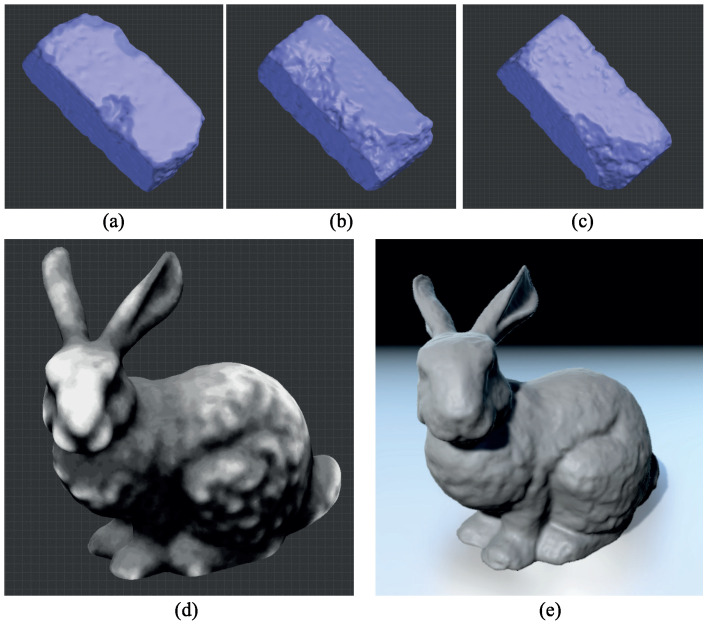
Weathered bunny model synthesized using three example bricks: (**a**–**c**) three example bricks; (**d**) generated depth map; (**e**) final result.

**Figure 12:**
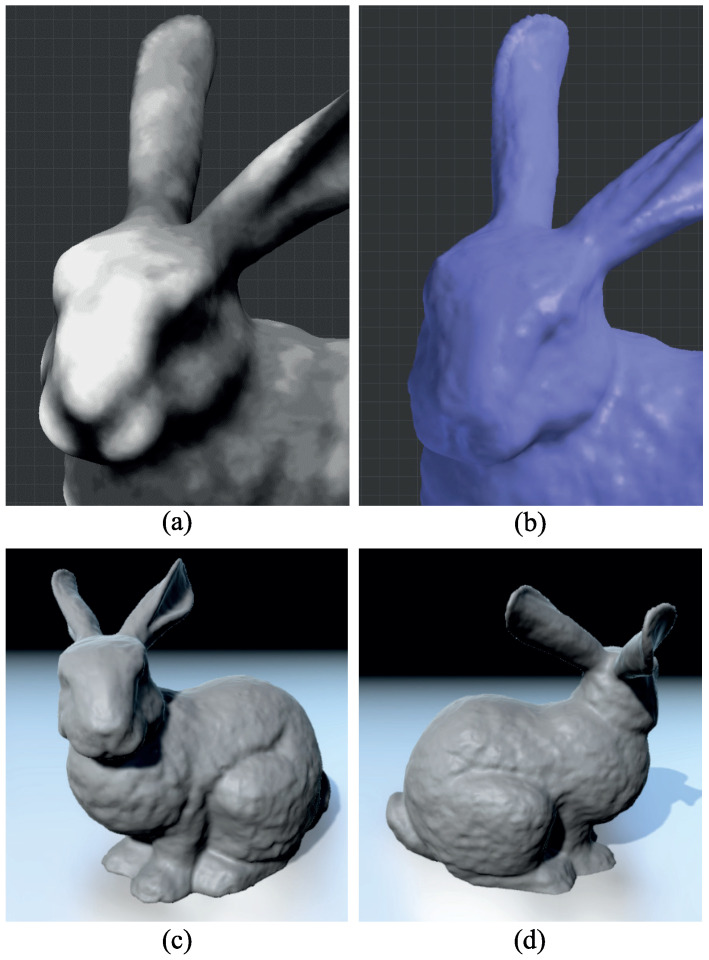
Weathered bunny model synthesized using five example bricks: (**a**) close-up of the generated depth map; (**b**) bunny model with applied depth map; (**c**,**d**) rendered results.

We tested our methodology on the Stanford bunny model to evaluate its effectiveness. Given that this approach is example-based, the quality of the synthesized weathering is intrinsically dependent on the example bricks used. Initially, we used a single example brick for this synthesis, as depicted in Figure 10. Due to the limited weathering patterns available for a single brick, the resulting weathered bunny model displayed relatively simple characteristics. However, as we increased the number of example bricks to three and five, the complexity of the weathering patterns improved, as illustrated in Figure 11 and Figure 12. Owing to the example-based approach of our method, the results appear more visually convincing than those from simulation-based methods such as in [11]. Additionally, leveraging 3D texture synthesis techniques, our proposed method is versatile enough to be applied to more complex surfaces, as evidenced by the bunny model showcased in our experiments. In contrast, simulation-based methods are primarily applied to planar surfaces, as these are easier to define within a simulation domain.

The Stanford bunny model comprises 40,000 vertices. To facilitate the synthesis, we resampled these vertices to achieve a uniform distribution across the surface. For the synthesis process, we sampled a 7×7 grid at each point on the surface, and for the guiding curvature and accessibility fields, we used a 3×3 grid of the same size.

## 5. Discussion

We introduced a novel method for simulating the effects of weathering on objects made of concrete using bricks as examples of weathered material. Through 3D scanning, we were able to precisely measure the extent of erosion on these samples. Our approach adapts and extends existing concepts from the *texture-by-numbers* and *texture synthesis on the surface* methodologies to 3D geometries. Specifically, we proposed the combined use of curvature and accessibility values to guide the synthesis process.

However, our work is not without limitations. First, our model assumes that weathering does not result in extremely concave shapes, such as those seen in cavernous weathering. This assumption limited our ability to apply depth maps from various face directions to measure erosion. Second, the use of depth maps is limited in terms of capturing significant shape changes, such as those resulting from breakage. In such instances, a volumetric representation of a shape, as demonstrated by Bhat and colleagues [25], could offer a more effective solution. Lastly, while using bricks as weathered examples enabled us to measure erosion, the simple geometry of bricks may not adequately capture the full range of interesting weathering patterns that might occur on more complex surfaces.

## Data Availability

The data presented in this study are available on request from the corresponding author.
